# Frailty in Cancer: Why Bother and Ways Forward

**DOI:** 10.1002/jcsm.70139

**Published:** 2025-12-08

**Authors:** Luka Cavka, Alessandro Laviano, Mitja Lainscak

**Affiliations:** ^1^ University Medical Centre Maribor Maribor Slovenia; ^2^ Department of Translational and Precision Medicine Sapienza University Rome Italy; ^3^ Faculty of Medicine University of Ljubljana Ljubljana Slovenia; ^4^ Division of Cardiology General Hospital Murska Sobota Murska Sobota Slovenia

**Keywords:** cancer, frailty, outcome

## 1 Frailty: From the Population at Large to Cancer Patients

Every era of medicine faces unique challenges. Today, aging is at the forefront, with the global population over 65 years expected to rise from 9% in 2019 to 17% in 2050. With this demographic shift, we must address the specific limitations of older adults, particularly frailty [1].

Frailty lacks a universal definition but broadly reflects a progressive decline in physiological and psychosocial reserves, resulting in vulnerability to stressors. The same insult—such as chemotherapy, infection or acute cardiopulmonary disease—may have very different outcomes in robust versus frail individuals [2, 3, 4, 5]. Reported prevalence varies widely due to inconsistent definitions, but studies suggest 11%–53% of elderly people are frail; the prevalence is higher in institutionalized elderly [2]. Cancer is also largely a disease of older adults: About 30% of new diagnoses occur in those aged 65–74 and 25% in those over 75 [6]. Some years ago, diagnosis of advanced cancer generally labelled patients with incurable disease; thus, work‐up beyond malignancy was considered irrelevant. However, over time, effective therapies emerged that transformed many types of cancer into chronic diseases. These therapies are not without side effects which are tolerated differently depending on the patient's general condition. We should avoid futile treatments in fragile patients where adverse effects are likely to outweigh potential benefits. With a lack of guidelines or expert consensus, these decisions are largely based on ad hoc individual clinical judgement.

Within this new framework of cancer management, comorbidities affecting the quality of life and physical performance are relevant [7, 8]. Physical performance and nutritional status‐related assessments are gaining traction while frailty still remains underrecognized. The standard tools like ‘Fried frailty phenotype’ (FFP) and ‘Comprehensive Geriatric Assessment’ (CGA) are time‐consuming and poorly suited to routine oncology practice.

Regarding open considerations about frailty in cancer patients, the work by Weinländer et al. [9] in the recent issue of the Journal is spot on. The authors meticulously evaluated the concordance of the considered gold standard but more time‐consuming tool FFP and clinically more convenient ‘Simple Frail Questionnaire’ (SFQ) [10, 11]. FFP assesses five criteria (low physical activity, poor endurance, unintentional weight loss, weakness and slowness), categorizing patients as robust, prefrail or frail [10]. SFQ uses a similar three‐level classification but is quick, requires no special training and can be administered remotely. Weinländer et al. found a strong correlation between both scores (*r*s = 0.65, *p* < 0.001) with good internal consistency (Cronbach's alpha coefficient 0.80). Evaluation by SFQ and FFP revealed that 41% and 31% of advanced cancer patients are prefrail, while 13% and 17% are frail, respectively. Comparing with the published meta‐analysis of frailty in cancer patients, they found a similar prevalence of prefrailty, while frailty was lower (43% and 42%, respectively) [12]. Both tools predicted mortality in multivariable analysis. These results demonstrate that SFQ is noninferior to FFP and provide a strong rationale for its clinical adoption. Weinländer et al. fill an important gap to demonstrate that SFQ is noninferior to FFP in frailty recognition or mortality risk reduction. These findings should guide clinical practice to promote frailty assessment which should improve our decision making for therapies that may cause more harm than benefit in frail patients.

## 2 Frailty in Cancer: Why Bother

**FIGURE 1:**
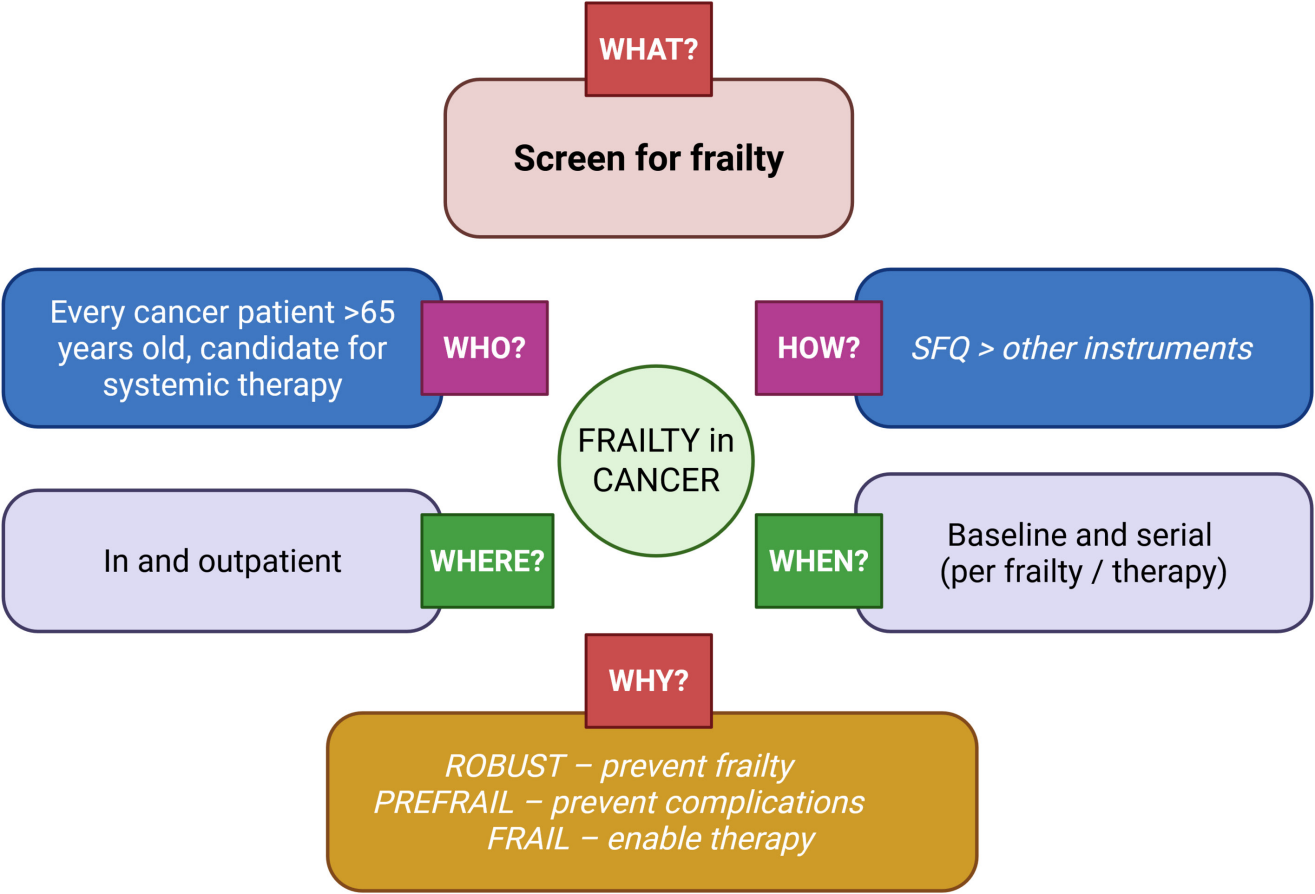
Management of cancer patients at risk of frailty (SFQ Simple Frail Questionnaire).

Frailty is not a risk factor for cancer itself but significantly shapes treatment feasibility and outcomes. A diagnosis of frailty should prompt clinicians to look for contributors, use frailty for risk stratification and incorporate frailty into therapy decisions. Universal screening for all cancer patients seems unrealistic, while targeted assessment of those starting therapy or showing disability should be feasible. Elements of frailty screening—such as nutritional and functional assessment—are already routine and should trigger interventions when abnormalities are found. Along with specific modalities, promotion of healthy lifestyle measures should be encouraged and other interventions if specific targets are identified (e.g., iron in iron deficiency and balance training in patients inclined to falls—Figure 1). Dyspnoea, occurring in about 21%–79% of patients with advanced cancer, often results from respiratory muscle loss and may benefit from respiratory physiotherapy [13].

**FIGURE 2:**
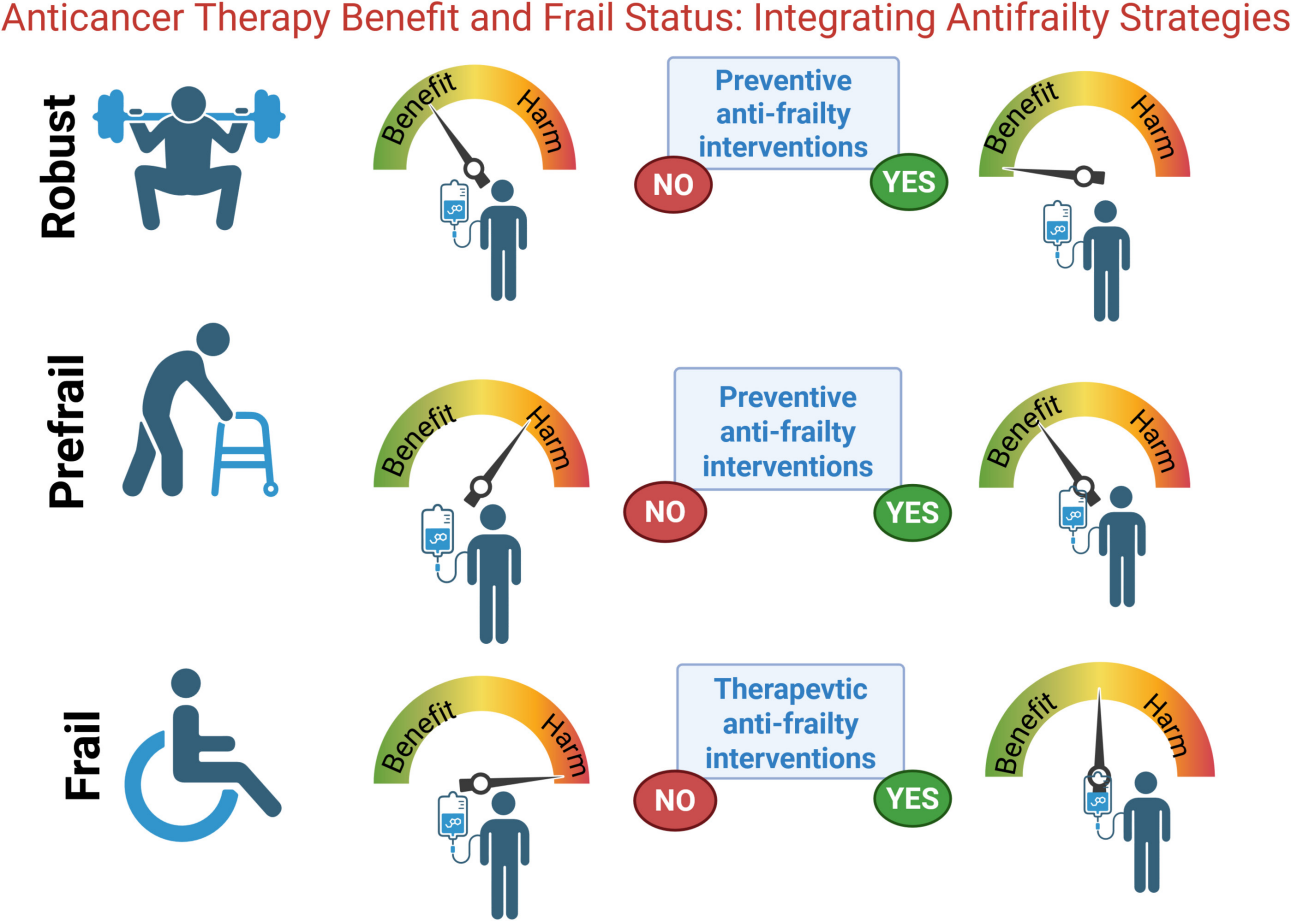
Anticancer therapy combined with antifrailty interventions in robust, prefrail and frail cancer patients.

Frailty is dynamic and potentially reversible, particularly in its early stages. To prevent frailty, it is crucial to commence interventions that increase intrinsic physiological capacity and psychosocial resilience (Figure 2). No matter if a patient is robust, prefrail or frail, the sooner we begin with antifrailty interventions, the higher the chances for better outcomes (Figure 2) [14, 15]. Effective interventions span multiple domains:

- *Physical activity* is central as it improves survival [16], muscle strength [17], balance, cardiovascular and mental health. Resistance and balance training reduce falls—the most common cause of acute frailty decompensation. Aerobic exercise, Tai Chi and multicomponent regimens also show benefit [18].
- *Nutrition* is equally important [19, 20]. Adequate protein and calorie intake, along with a Mediterranean‐style diet, support muscle mass and resilience [21]. Sarcopenia and cachexia often overlap with frailty, and interventions targeting these states improve survival and quality of life [22, 23].
- *Combined strategies* have shown promise. The SPRINTT trial demonstrated that supervised and at‐home exercise with nutritional counselling slowed functional decline compared with education alone [24].
- *Rehabilitation* in advanced cancer improves quality of life, fatigue, strength and aerobic fitness [25, 26]. Intensive exercise may be unrealistic in severely frail patients, where transcutaneous electrical muscle stimulation may be beneficial.

## 3 Frailty in Cancer: Ways Forward

At present, decisions about systemic therapy in frail cancer patients largely rely on clinical judgement, without formal guidelines. Physicians depend heavily on patient‐reported activity levels, which may be unreliable. New technologies—wearable sensors and mobile apps—offer objective monitoring [4], while emerging biomarkers (e.g., 3‐methylhistidine, interleukins and micronutrients) could support assessment [27]. The value of frailty screening extends beyond risk stratification: It identifies targets for intervention and integrates supportive care into oncology. A new concept in the management of patients with cancer is emerging suggesting that senescence, as assessed by biological age, could represent a comprehensive, treatable and sensitive marker of poor resilience, encompassing the concepts of frailty, malnutrition and cachexia [28].

In this context, Weinländer et al. provide compelling evidence that the SFQ is a valid, practical tool for frailty assessment in cancer patients. Within minutes, it yields clinically relevant information that should guide both therapy decisions and supportive interventions. Incorporating frailty assessment into oncology practice will help avoid harmful treatments in vulnerable patients, tailor supportive strategies and ultimately improve outcomes.

## Funding

M.L. is supported by Slovenian Research and Innovation Agency (Grant No. P03‐456).

## Conflicts of Interest

The authors declare no conflicts of interest.
